# Universal Access to Surgical Care and Sustainable Development in Sub-Saharan Africa: A Case for Surgical Systems Research: Comment on "Global Surgery – Informing National Strategies for Scaling Up Surgery in Sub-Saharan Africa"

**DOI:** 10.15171/ijhpm.2018.106

**Published:** 2018-10-29

**Authors:** Emmanuel M. Makasa

**Affiliations:** ^1^Republic of Zambia, Ministry of Foreign Affairs, Lusaka, Zambia.; ^2^School of Medicine, Faculty of Health Sciences, University of Witwatersrand, Johannesburg, South Africa.

**Keywords:** Surgery, Health Policy, Implementation Research

## Abstract

National level experiences, lessons learnt from the Millennium Development Goal (MDG) era coupled with the academic evidence and proposals generated by the Lancet Commission on Global Surgery (LCoGS) together with the economic arguments and recommendations from the World Bank Group’s "Essential Surgery" Disease Control Priorities (DCP3) publication, provided the impetus for political commitments to improve surgical care capacity at the primary level of the healthcare system in low- and middle-income countries (LMICs) as part of their drive towards universal health coverage (UHC) in the form of World Health Organization (WHO) Resolution A68.15. This global commitment from governments must be followed up with development of a Global Action Plan and a global coordination mechanism supported by regional implementation frameworks on the part of the WHO in order for the organisation to better coordinate all stakeholders and sustain the technical support needed to develop and implement national surgical health policy in the form of National Surgical Obstetric and Anaesthesia Plans (NSOAPs). As expounded by Gajewski et al, data and research output on surgical care is essential to informing policy development and programme implementation. This area still remains a challenge in sub-Saharan Africa (SSA) but it is envisaged that countries will include this key component in their ongoing national surgical healthcare policy development and programme implementation. In the Zambian case study, research in the area of Global Surgery investment-the surgical workforce scale-up is used to demonstrate the important role of implementation research in the development and implementation of the Zambian NSOAP as well as the need for international collaborations to this end. Scale-up reviews informed by implementation research to evaluate progress on the commitments contained in Resolution A68.15 and Decision A70.22 are essential to sustain the momentum and to help maintain focus on the gaps in all countries. There are opportunities for non-state actors especially local sub-regional academic institutions, non-governmental organizations (NGOs) and private sector to play a key role in surgical healthcare policy development and implementation research. Collection of and better information management of standardised surgical care indicators is essential for such research, for bi-annual WHO progress reporting and for demonstration of impact to justify and encourage further investments in surgical care.

While the effort to advance access to safe and affordable surgery and anaesthesia care may be as old as the two domains themselves, it is not far from the truth for one to state that it’s only in the recent past that surgery and anaesthesia care have seen the light of day in as far as being part of the global public health and development agenda is concerned. With the 2015 transition from the silo creating and disease specific global Millennium Development Goals (MDGs); One of whose pitfalls was the lack of integration across sectors^1^ that supported disease specific health programmes at country level, to the multi-sectoral, multi-disciplinary, integrated and cross-cutting partnership approach towards sustainable development whose target is set at 2030,^1,2^ health still remains at the core of the new global development agenda.^3^

Member States (MS) and None State Actors^4^ (NSAs) have recognised and endorsed Surgery and Anaesthesia as components of universal health coverage (UHC) and therefore essential to creating the appropriate healthy environment for global sustainable development with the passing of the World Health Organisation’s (WHO’s) resolution WHA68.15^5^ in the year 2015 and decision WHA70(22)^6^ in 2017. This was in addition to the World Bank Group’s publication of a full volume on “Essential Surgery”^7^ in the third edition of their Disease Control Priorities (DCP3) and the Lancet Commission on Global Surgery’s (LCoGS’s) “*Global Surgery 2030: evidence and solutions for achieving health, welfare, and economic development*”^8^ report; both of which were published the same year, 2015.

In order to translate global political commitments into national health policy and programmes that can further translate into tangible health service delivery in this regard, 3 years down the line since resolution WHA68.15; many low- and middle-income countries (LMICs) in sub-Saharan Africa (SSA) are embarking on the development of their National Surgical Obstetric and Anaesthesia Plans^9^ (NSOAPs) as the vehicle to improving universal access to safe and appropriate surgical and anaesthesia care within their primary health system (at the District Hospital).

As expounded by Gajewski et al, the Republic of Zambia was the first to pilot the LCoGS’s blueprint^10^ for a systems approach to translating the political commitments contained in resolution WHA68.15 into national health policy in the form of an NSOAP and therefore makes a good case to study though my opinion on this issue may be considered subject to bias with me being a Zambian national by some, while others may still argue that my nationality makes me a good commentator on this case study.

The identification by the authors of surgical health systems research especially at District as an important gap^10^ that needs closing in order to inform the implementation of these surgical obstetric and anaesthesia country plans is fully acknowledged and supported by evidence from the inception of the Zambian NSOAP^11^ development process which is also the case in many other countries embarking on this process.

A Zambia nationwide surgical health system situation analysis,^12-14^ though done prior to the NSOAP process, was essential and not only provided the evidence that spurred the country into diplomatic action to spearhead the development of global surgical care health policy at the United Nations in Geneva and Vienna, but also provided some of the baseline information used in the formulation of not only the Zambian NSOAP but also the surgical health systems research grant applications of COST-AFRICA and SURG-AFRICA that the authors use in this case study.

Because of the very limited data from scientific studies in the fields of Surgery and Anaesthesia,^15^ worse in surgical health systems, in the SSA region, that could have better informed the development of NSAOPs, its hardly wrong to assume that a national surgical obstetrics and anaesthesia planning process that is only informed by a snap cross-sectional situational analysis could be flawed.

For this reason, the Zambian NSOAP development process was further supported by surgical health systems data obtained from other processes that included expert opinions, stakeholder engagements in focused group discussions for priority setting, drafting and validation as well as costing.

The outcome Zambian NSOAP, launched at the 70th World Health Assembly Global Surgery Side-Event in Geneva, also includes surgical health systems research and quality improvement as one of the pillars of an effective surgical, obstetric, and anaesthesia health systems.

It is therefore envisaged that the Zambian universal access to safe surgery and anaesthesia capacity building process, that is being guided by the Zambian NSOAP implementation at the district hospital facility, will continue to be improved based on information generated from local and hopefully regional surgical obstetrics and anaesthesia plan implementation research.

While funding is highlighted as the main constraint to surgical health systems research output in the Zambian NSOAP, limited research skills, capacity and culture could be the other inhibiting factors that could be addressed through international research collaborations as evidenced by both the COST-Africa^10^ and SURG-Africa^10^ research grants that have provided both the funding and research training programs.

The absence of large scale, let alone multi-disciplinary or multicentre user friendly surgical health information systems or databases is another factor in the way of surgical health systems research when one considers the disjointed paper-based surgical patient health information and low surgical case volume recorded in most health facilities in Zambia. It is my sincere hope that innovative use of mobile phone technology can help us address this challenge in view of the fact that Zambia currently has a relatively high mobile phone penetration (79.07%)^16^ and coverage.

The Republic of Zambia is also in the process of establishing a WHO Regional Collaboration Centre (RCC) on surgical health information. This centre will also serve as a key driver for surgical health systems research output for Zambia and the 16 MS of the Southern African Development Community (SADC) so that our future surgical health policy formulations, surgical programme development, and surgical service delivery are well informed and evidence-based.

Other global and regional gaps that could affect national surgical and anaesthesia policy, programme and service delivery as per resolution WHA68.15 may include, among others, the lack of a robust global surgery governance and coordination mechanism that could mobilise appropriate resources, support surgical health systems research and provide a platform for sharing of best practice for LMICs in SSA and other parts of the developing world. A SSA regional implementation framework could also be essential for benchmarking for both NSOAP development as well as implementation. The WHO which is the natural lead in this area unfortunately continues to lag behind in its financial and technical capacity to support MS in their quest to improve universal access to safe and affordable surgical care. In such a scenario, the role of none-state actors becomes even more important and works best when they are well coordinated regionally or at country level with the national Ministry of Health taking the lead role as was the case in the COST-Africa study and the Zambian NSOAP development process. We continue to engage and work with regional inter-governmental organisations that are new to public health work such as the SADC, the East Central and Southern Africa Health Community (ECSA-HC) and indeed the African Union (AU) with the hope that they too can take up this challenge as they embrace public health work for development with the spirit of “leaving no one behind.” It is my sincere hope that surgical health systems research will not be left behind as we formulate strategies for scaling up surgery in SSA.

## Ethical issues

Not applicable.

## Competing interests

Author declares that he has no competing interests.

## Author’s contribution

EMM is the single author of the paper.
